# Effect of Folic Acid Intake on Infant and Child Allergic Diseases: Systematic Review and Meta-Analysis

**DOI:** 10.3389/fped.2020.615406

**Published:** 2021-01-18

**Authors:** Zekun Chen, Yan Xing, Xue Yu, Yuqi Dou, Defu Ma

**Affiliations:** ^1^School of Public Health, Peking University Health Science Center, Beijing, China; ^2^Department of Pediatrics, Peking University Third Hospital, Beijing, China

**Keywords:** folic acid intake, folate status, infant and child, allergic diseases, meta-analysis

## Abstract

**Objective:** This study aimed to analyze the effect of folic acid supplements on infant and child allergic diseases through systematic review and meta-analysis.

**Design:** PubMed, The Cochrane Library and references of related articles published before January 1, 2020 were searched.

**Setting:** Meta-analysis was used to explore the influence of folic acid on skin allergies (eczema, and atopic dermatitis) and respiratory allergies (asthma, wheezing, and allergic rhinitis).

**Participants:** Data were collected from 15 studies with 244,018 individual participants from five different countries for meta-analysis.

**Results:** Folic acid was confirmed as a risk factor for allergic diseases in infant and child. The risk of allergic diseases dramatically increased when maternal folic acid intake <400 μg/day (RR = 1.050; 95% CI = 1.027–1.073) during pregnancy. Stratified analyses revealed that the association was significant only for respiratory allergy (RR = 1.067; 95% CI = 1.028–1.108) and pregnant women who only used folic acid supplements (RR = 1.070; 95% CI = 1.030–1.112) and that countries without folic acid fortification (RR = 1.046; 95% CI = 1.026–1.067).

**Conclusions:** This study suggested that folic acid intake can be a risk factor for allergic diseases, especially respiratory tract allergies among infants and young children. Furthermore, pregnant women should pay attention to supplementation of folic acid from both folic acid supplements and fortified foods with folic acid during pregnancy.

## Introduction

Allergic diseases involve multiple organs and systems (1) and are one of the most serious public health problems worldwide. World Health Organization indicated that high prevalence rate of allergic diseases ranked sixth place worldwide. More than 20% of world population has suffered from it, and the incidence rate is still increasing (2). Moreover, the worldwide incidence of allergic diseases of infant and child is increasing with the development of social economy, the change of diet structure, and the influence of environmental and genetic factors; thus, allergic diseases are the most common chronic diseases not only for adults but also for infants and children (3). During the infancy period, allergic diseases mainly include repeated skin lesions (eczema and atopic dermatitis), pruritus, respiratory symptoms (rhinitis, rhinorrhea, and asthma), gastrointestinal symptoms (abdominal pain, diarrhea, and hematochezia), and sleep disorders. These symptoms can affect the metabolism, nutritional absorption, and intellectual and physical activities of the infant and child and adversely influence their physical and mental health. Therefore, possible factors that affect allergic diseases risk should be determined.

Folate, including naturally occurring folate and synthetic folic acid, is a 1-carbon source crucial to DNA and RNA replication during cell division and methylation of DNA, histones, and other proteins (4). Folate can not be synthesized by human body, so it depend on external supplementation to maintain normal levels. Naturally occurring folate is found in a wide variety of foods, however, natural folate has a high loss rate during cooking and preservation due to its instability (5). External supplementation of folate may occur as synthetic folic acid or 5-methyltetrahydrofolate (5-MTHF). Synthetic folic acid (pteroylmonoglutamic acid) has no biological functions unless it is reduced to dihydrofolate and tetrahydrofolate. Moreover, long-term high doses of synthetic folic acid may interfere with the action of other drugs, interfere with the absorption of other nutrients, or mask the symptoms of vitamin B12 deficiency. 5- MTHF is a bioactive form of folic acid and it is the main form of folic acid in the human blood. It can be directly absorbed and used by the human body. It is now thought that naturally occurring 5-MTHF, which is more effective than folic acid in improving folate status, may have important advantages over synthetic folic acid, so supplementation with 5-MTHF may be an effective substitute for folic acid (6–8). Previous animal studies suggested that maternal folate deficiency can change the ultrastructure of lobus frontalis in fetal rats. This condition may lead to abnormality of the neuronal function and disturb the fetal brain development. Clinical studies indicated that folate deficiency is also associated with weight loss, slow growth in children, and megaloblastic anemia (9).

However, recent studies found that folic acid functions as a methyl donor in biochemical reactions, and a methyl donor-rich diet may increase the risk of acquiring allergic diseases through DNA methylation (10). Haberg et al. (11) reported that folic acid supplementation to pregnant women in the early stages might lead to a small increased risk for wheezing in the offspring during the first 18 months of life. Dunstan et al. (12) revealed that children aged 1 with eczema often have an increased folic acid exposure history. Bekkers et al. (13) conducted a birth cohort study among 3,786 children and found that maternal folic acid supplementation is a risk factor for wheezing only in children under 1 year of age. However, no significant association was observed between maternal folic acid supplementation and wheezing in children over 1 year of age. Although many studies focusing on the effects of early folate use on allergic diseases have been conducted, the results are conflicting.

This study aimed to analyze the effect of folic acid supplements on infant and child allergic diseases via systematic review and meta-analysis. Moreover, stratified analysis and meta-regression analysis were performed to clarify the underlying pathways behind the association between folic acid supplements and allergic diseases.

## Materials and Methods

### Study Protocol and Search Strategy

This study protocol was in accordance with the meta-analysis of observational studies in epidemiology (14).

Under the guidance of professional librarian, literature search was conducted in PubMed and The Cochrane Library for English articles. All studies were published before January 1, 2020. MeSH terms for literature extraction from online resources were as follows: (folate OR folacin OR “folic acid” OR “vitamin B9”) AND (“Asthma” OR “Bronchial Hyperreactivity” OR “Respiratory Sounds” OR asthma* OR respiratory OR wheeze* OR “reactive airway” OR atopy OR allergy). List of references of related reviews and articles were also checked. Moreover, we contacted the authors of articles via e-mail when the required data was not reported in the articles to ensure that all necessary data were obtained.

### Inclusion Criteria

Studies for the meta-analysis were selected based on the following a-priori-defined inclusion criteria: (1) Studies designed as cohort study; (2) Studies with the control group; (3) Available relative risk (RR) and hazard ratio with 95% confidence interval (CI) on folic acid intake and allergic diseases; (4) The incidence rate of allergic diseases or the cumulative incidence rate is taken as the outcome index. Allergic diseases include any allergic diseases, such as skin allergies and respiratory allergies. “Any allergic disease” refers to those mentioned in the article but with no specific classification of allergic diseases. Skin allergies include eczema and atopic dermatitis, and respiratory allergies comprise asthma, wheezing, and allergic rhinitis. Studies that provided only rough estimates were excluded. If study samples overlap in at least two publications, or if multiple publications describe the same study aspects, then only the publication with the largest sample was considered.

### Data Extraction

Data were extracted using a standardized spreadsheet independently by two reviewers (Yan Xing and Zekun Chen) on a prespecified form. A third author (Defu Ma) was consulted when discrepancies occurred. Studies were selected and read carefully one by one. Standard data extraction tables were used to extract specific information of each study, including the first author, publication date, research location, follow-up age, folic acid exposure dose, intake duration, types of a–llergic diseases, the number of people in the exposed group and control group, and RR values or hazard ratios and its CI. All the RR values extracted in this study were RR values adjusted by possible confounding factors such as feeding, family history.

### Quality Assessment

Newcastle–Ottawa Scale (NOS) (15) was used to evaluate the quality of included studies, and the assessment consisted of nine items in three parts: (1) selection, in which each item can obtain at most one star; (2) comparability, in which each item can attain at most two stars; and (3) exposure, in which each item can acquire at most one star. Total points are 1–9 stars. Grade ≥ 6 stars was regarded as high-quality research (A-grade), and grade between 1 and 5 stars was considered low quality research (B-grade). The stars were assigned for studies that reported follow-up of at least 4 years with missed follow-ups of <25%.

### Statistical Analysis

The heterogeneity among results was analyzed by Q statistic. No heterogeneity will be defined if Q statistic follows the chi-square distribution and *P* > 0.1. I^2^ statistic, which can be used to quantitatively represent the between-study heterogeneity, was also calculated. Fixed effect model is adopted if there is no heterogeneity; otherwise, the random-effects model is performed. Forest plots were generated for pooled analysis, and weighted mean differences (WMDs), 95% CIs and *P*-values were reported. Stratified analysis by follow-up age, folic acid exposure dose, supplementation duration of folic acid, geographic region, supplementation type, and disease was conducted according to the different characteristics of the studies. Meta-regression analyses were conducted to assess whether the effect of folic acid supplements on allergic diseases were related to folic acid exposure dose. Sensitivity analysis was employed to investigate the influence of a single trial on the overall effect as estimated by omitting one study in each turn. Publication bias was assessed with funnel plots, Egger's linear regression test or Begg's rank correlation test. All statistical analyses were performed with STATA (version 12; Stata Corp., College Station, TX, USA). *P* < 0.05 was considered statistically significant unless otherwise specified.

## Results

### Document Retrieval Results

**Figure 1:**
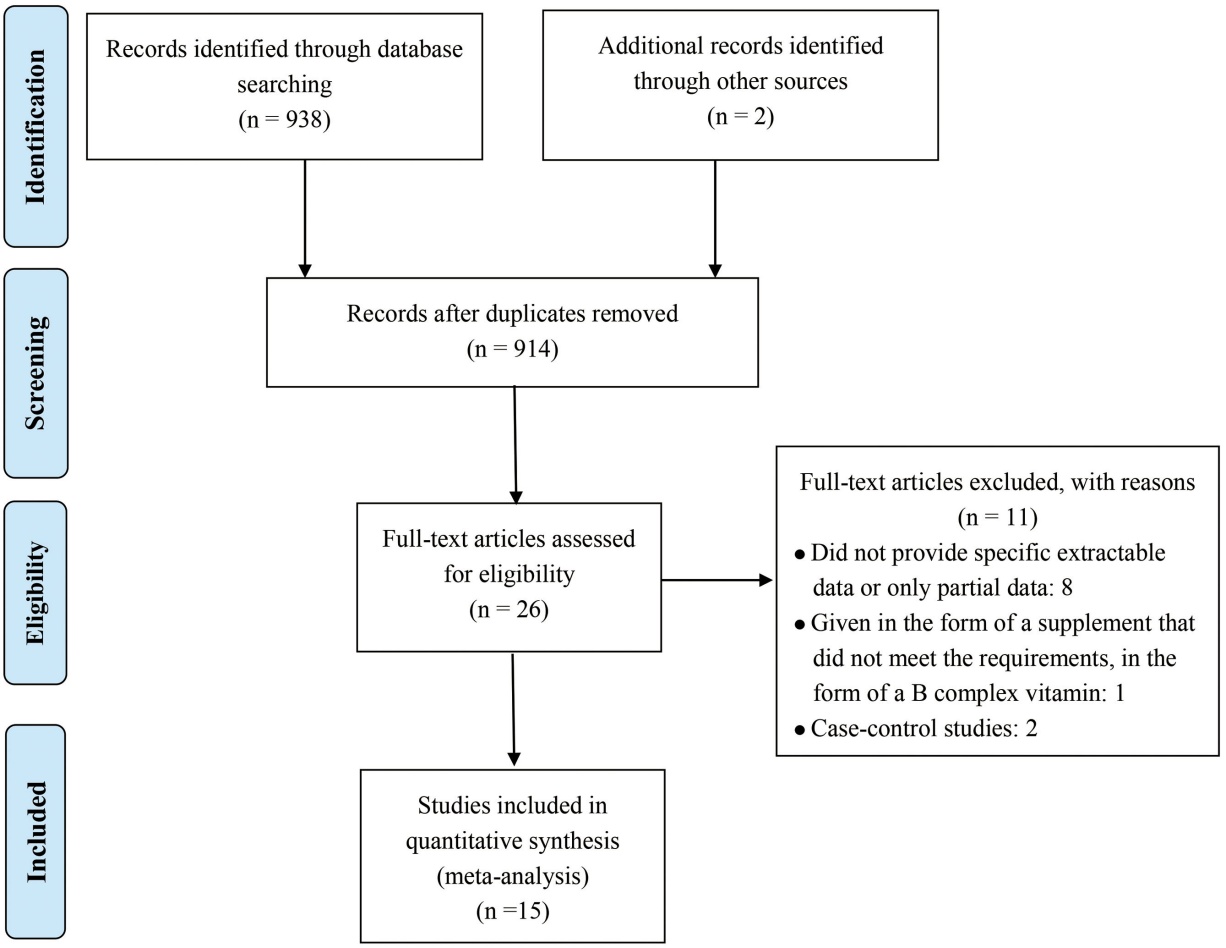
Flow diagram of study selection process.

Figure 1 shows the detailed inclusion of qualified reports involving folic acid and allergic diseases. A total of 938 full texts were found according to the MeSH terms. Among which, 914 records were excluded according to zoological reviews depending on the title, abstract, or keyword. Two additional records were obtained from the references. Finally, 26 full-text studies were screened for detailed evaluation. Among which, eight studies were excluded for not providing sufficient extractable data, and one was excluded for not giving supplements in a satisfactory form. Two others were excluded because they were case–control studies. Finally, 15 cohort studies were included (11–13, 16–27).

### Basic Features Included in the Study

Table 1 presents the basic features of the 15 included studies with 244,018 analyzed cases. Five were conducted in Netherlands, four in the United States, three in Australia, two in Norway, and one in the United Kingdom. All 15 articles were cohort studies published in 2008 and beyond, with the latest published in 2019. Folic acid exposure period was distributed among different stages of pregnancy (including early, mid, late, or throughout the pregnancy). Exposure dose ranged from 250 μg/day to 1,238 μg/day. Follow-up age of infant and child was 1–9.7 years old. Each included study was assigned scores according to the NOS, and Table 2 shows the results. All studies were of high quality because they had scores from 7 to 9 with the mean score of 8.40. Star was deducted where follow-up was <4 years (11, 12, 17, 25, 27) and missed follow-ups >25% (12, 16, 20, 25).

**Table 1:** Summary of eligible studies.

**References**	**Country**	**Sample size**	**Follow-up age**	**Folic acid exposure dose**	**Folic acid exposure period**	**Allergic outcomes**
Granell (16)	UK	5,364	7.5 years	No report	At 18 weeks, 32 weeks	Asthma, wheeze
Haberg (11)	Norway	32,077	6–18 months	400 ug recommended	First trimester, after first trimester	Wheeze
Whitrow (17)	Australia	490	3.5 years	No report for prepregnancy, median 700 ug/day for early pregnancy, median 300 ug/day for late pregnancy	Prepregnancy, early pregnancy (<16 weeks), late pregnancy (30–34 weeks)	Asthma
Magdelijns (18)	Netherlands	2,834	2 years, 6–7 years,	400 ug/day	Early pregnancy (4–8 weeks) whole pregnancy, another period	Eczema, AD, wheeze, asthma
Martinussen (20)	America	1,499	6 years	Average 497 ug/day	Prepregnancy, first trimester	Asthma
Kiefte-de Jong (19)	Netherlands	8,742	4 years	400–500 ug/day	Prepregnancy, early pregnancy(<10wk)	Wheeze, AD
Bekkers (13)	Netherlands	3,786	1–8 years	no report	During pregnancy	Asthma, wheeze, eczema
Dunstan (12)	Australia	484	1 years	Average 250 ug/day or 500 ug/day	During pregnancy	Any allergic disease, wheeze, eczema
Zetstra-van der Woude (21)	Netherlands	35,604	9.5 years	500 ug/day	During pregnancy.	Asthma
Veeranki (22)	America	104,428	4.5–6 years	No report	First trimester, after first trimester, during pregnancy	Asthma
Parr (23)	Norway	39,846	7 years	400–600 ug/day	At 22 weeks	Asthma
Dekker (24)	Netherlands	5,653	10 years	400–500 ug recommended	Prepregnancy, start 0–10 weeks, start >10 weeks, whole pregnancy	Asthma
Roy (25)	America	858	3 years	400–800 ug/day	Second trimester, third trimester	Wheeze, AD
Trivedi (26)	America	1,279	7–10 years	Average 930 or 1238 ug/day	First trimester, second trimester	Asthma
Molloy (27)	Australia	1,074	1 years	Average 750 or exceed 1,000 ug/day	First trimester, second trimester	eczema

*AD, atopic dermatitis*.

**Table 2:** Quality assessment of included studies in the meta-analysis using the Newcastle-Ottawa scale.

**References**	**Selection**	**Comparability**	**Outcome/exposure**	**Total score**
Granell (16)	****	**	**	8
Haberg (11)	****	**	**	8
Whitrow (17)	****	**	**	8
Magdelijns (18)	****	**	***	9
Martinussen (20)	****	**	**	8
Kiefte-de Jong (19)	****	**	***	9
Bekkers (13)	****	**	***	9
Dunstan (12)	****	**	*	7
Zetstra-van der Woude (21)	****	**	***	9
Veeranki (22)	****	**	***	9
Parr (23)	****	**	***	9
Dekker (24)	****	**	***	9
Roy (25)	****	**	*	7
Trivedi (26)	****	**	***	9
Molloy (27)	****	**	**	8

### Outcomes of Meta-Analysis

**Figure 2:**
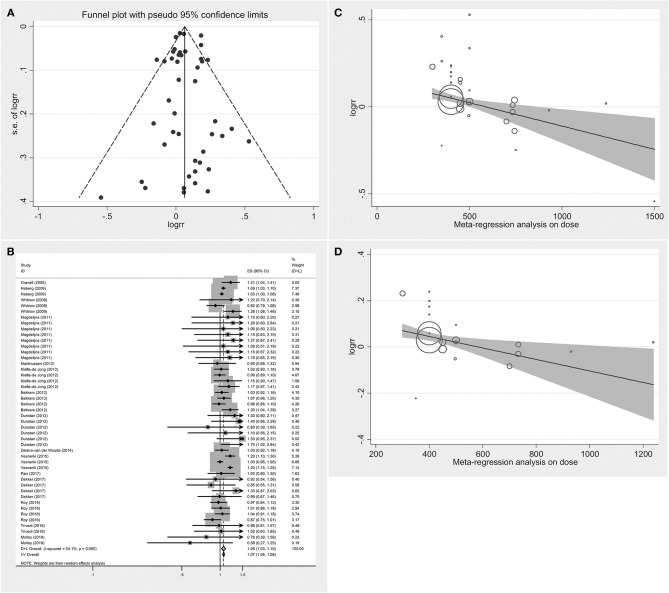
Summary analysis **(A)** Funnel plot analysis of the effect of folic acid intake on infant and child allergic diseases. **(B)** Forest diagram analysis of the effect of folic acid intake on infant and child allergic diseases. **(C)** Meta-regression analysis on dose of the effect of folic acid intake on infant and child allergic diseases. **(D)** Meta-regression analysis on dose of the effect of folic acid intake on respiratory allergic diseases.

Figure 2A displays the funnel plot of the effect of folic acid supplements on allergic diseases in infant and child. Although the funnel plot had values beyond the funnel boundary, its distribution was basically symmetric. Meanwhile, Begg's test and Egger's test did not find publication bias. *P*-values of the two tests were 0.379 and 0.900, respectively. Therefore, meta-analysis can be performed.

Figure 2B illustrates the pooled analysis of the effects of folic acid on allergic diseases. Compared with control group, the risk was significantly increased in the folic acid supplement group (RR = 1.064; 95% CI: 1.028–1.101). Sensitivity analyses with each individually excluded study suggested that no individual study had significant influence on the pooled results. Table 3 shows the stratified analysis results for the effects of folic acid intake on allergic diseases. Follow-up age was divided into the <4 (RR = 1.044, 95% CI = 1.023–1.064) and ≥4-year-old groups (RR = 1.075, 95% CI = 1.013–1.141), both of them were significant. In the stratification analysis of folic acid exposure dose, significant association was found only when the dose was <400 μg/day (RR = 1.050; 95% CI = 1.027–1.073). Moreover, meta-regression analyses (Figure 2C) showed that the risk effect of folic acid supplements on allergic diseases decreased with the increasing of folic acid exposure dose (*r* = −0.002, *p* = 0.015). Folic acid exposure period was distributed to three groups (First trimester vs. After first trimester vs. Whole pregnancy). The subgroup analysis suggested that folic acid supplementation during the whole pregnancy group significantly increased 12% allergic disease risk (RR = 1.124; 95% CI = 1.091–1.157).

**Table 3:** Stratified analysis of the effects of folic acid on allergic diseases.

**Stratified analysis**	**Heterogeneity test**				**Pooled RR values (95%CI)**	**Pooled RR values' tests statistic**	
	**q**	**d.f**.	**p**	**I^**2**^**		**Z**	**P**
**Follow-up age, year**							
<4	36.16	25	0.069	30.9%	1.044 (1.023~1.064)	2.42	0.015
≥4	50.43	19	0.000	62.3%	1.075 (1.013~1.141)	2.39	0.017
**Folic acid exposure dose, μg/day**							
≤400	12.47	14	0.569	0.0%	1.050 (1.027~1.073)	4.39	0.000
>400	17.91	19	0.528	0.0%	1.005 (0.964~1.048)	0.25	0.805
**Supplementation of folic acid by period**							
First trimester	16.96	13	0.201	23.3%	1.068 (1.037~1.100)	1.85	0.038
After first trimester	23.92	11	0.013	54.0%	1.049 (0.997~1.103)	1.85	0.064
Whole pregnancy	36.09	19	0.010	47.4%	1.124 (1.091~1.157)	2.67	0.008
**Supplementary type**							
Supplements + fortified foods	30.88	18	0.030	41.7%	1.042 (0.990~1.098)	1.30	0.194
Supplements	66.35	26	0.000	60.8%	1.070 (1.030~1.112)	3.46	0.001
**Geographic region**							
Countries without folic acid fortification	16.78	24	0.858	0.0%	1.046 (1.026~1.067)	4.61	0.000
Countries with folic acid fortification	68.04	18	0.000	73.5%	1.074 (0.999~1.155)	1.92	0.055
**Disease**							
All allergic diseases	0.04	1	0.833	0.0%	1.348 (0.956~1.902)	1.70	0.089
Skin allergy	17.79	12	0.122	34.6%	1.021 (0.959~1.087)	0.93	0.352
Respiratory allergic	76.52	30	0.000	60.8%	1.067 (1.028~1.108)	3.41	0.001

Interestingly, subgroup analysis showed that when pregnant women used both folic acid supplements and fortified foods with folic acid, the risk was not significant. The United States (20, 22, 25, 26), Australia (12, 17, 27), and the United Kingdom (16) have implemented folic acid fortification programs that mandate fortification of grains and wheat flour. However, apart from the UK, none of the countries in Europe, had started folate fortification programs (11, 13, 18, 19, 21, 23, 24). To explore the differences, we conducted a subgroup analysis of countries with or without folic acid fortification. The Stratified analysis by geographic region revealed that countries without folic acid fortification (RR = 1.046; 95% CI = 1.026–1.067) presented a significant risk effect of folic acid on allergic diseases.

The RRs were 1.348 (95% CI = 0.956–1.902), 1.021(95% CI = 0.959–1.087), and 1.067 (95% CI = 1.028–1.108) for the all allergic disease group, the skin allergy group, and the respiratory allergic group, respectively. Given that folic acid exposure was only a risk factor for respiratory allergies and had no significant effect on the incidence of skin allergies and all allergic diseases, we performed a stratified analysis for that included only respiratory allergic diseases (Table 4). Subgroup analyses for respiratory allergies revealed similar results with that for allergic diseases. Folic acid exposure caused a significant risk on respiratory allergic diseases only for that the folic acid exposure dose was <400 μg/day (RR = 1.048; 95% CI = 1.026–1.071) and that when pregnant women only used folic acid supplements (RR = 1.070; 95% CI = 1.025–1.117) and that countries without folic acid fortification (RR = 1.046; 95% CI = 1.026–1.067). Moreover, meta-regression analyses (Figure 2D) showed that the risk effect of folic acid supplements on respiratory allergic diseases decreased with the increasing of folic acid exposure dose (*r* = −0.002, *p* = 0.054). In addition, the subgroup analysis suggested that folic acid supplementation after first trimester group (RR = 1.066; 95% CI = 1.011–1.124) had statistically remarkable relevance for respiratory allergic diseases risk. Since folic acid intake is a risk factor for allergic diseases only when the dose was <400 μg/day, we performed subgroup analysis for the studies with the folic acid exposure dose <400 μg/day or more than 400 μg/day, respectively. (data not shown). It showed that the folic acid exposure dose more than 400 μg/day was not a risk factor for all allergic diseases.

**Table 4:** Stratified analysis of the effects of folic acid on respiratory allergic diseases.

**Stratified analysis**	**Heterogeneity test**				**Pooled RR values** **(95%CI)**	**Pooled RR values' tests statistic**	
	**q**	**d.f**.	**p**	**I^**2**^**		**Z**	**P**
**Follow-up age, year**							
<4	16.91	11	0.110	35.0%	1.043 (1.022~1.064)	2.26	0.024
≥4	46.05	18	0.000	60.9%	1.086 (1.021~1.155)	2.61	0.009
**Folic acid exposure dose, μg/day**							
≤400	8.97	8	0.345	10.8%	1.048 (1.026~1.071)	3.38	0.001
>400	1.83	11	0.999	0.0%	0.995 (0.947~1.046)	0.20	0.845
**Supplementation of folic acid by period**							
First trimester	13.68	9	0.045	34.2%	1.064 (0.987~1.147)	1.63	0.103
After first trimester	18.84	9	0.027	52.2%	1.066 (1.011~1.124)	2.35	0.019
Whole pregnancy	24.91	10	0.006	59.9%	1.060 (0.986~1.141)	1.58	0.115
**Supplementary type**							
Supplements + fortified foods	13.94	10	0.176	28.3%	1.060 (0.995-1.129)	1.33	0.184
Supplements	62.51	19	0.000	69.6%	1.070 (1.025~1.117)	3.09	0.002
**Geographic region**							
Countries without folic acid fortification	13.07	17	0.731	0.0%	1.046 (1.026~1.067)	4.47	0.000
Countries with folic acid fortification	51.51	12	0.000	76.7%	1.070 (0.986~1.160)	1.62	0.104

## Discussion

This meta-analysis supported a causal link between the use of folic acid supplements during pregnancy and the increased risk of respiratory allergic diseases in infants and young children. However, the risk of allergic diseases dramatically increased only for that the folic acid exposure dose was <400 μg/day and that when pregnant women only used folic acid supplements and that countries without folic acid fortification. Moreover, meta-regression analyses showed that the risk effect of folic acid supplements on respiratory allergic diseases decreased with the increasing of folic acid exposure dose.

Folate affects the development of childhood asthma possibly through the methylation of variable DNA in the mother's uterus. During DNA methylation, a methyl group is transferred from s-adenosylmethionine to cytosine by the action of a transmethylase, which regulates cell growth (28). Hollingsworth et al. (29) observed that transgenic mice supplemented with diet consisting of methyl donors, including folic acid, choline, l-methionine, and betaine will produce progeny with airway, hyperactivity, inflammatory response, changes in DNA methylation patterns, and reduced gene expression compared with the mice with a low methyl donor diet. In our present meta-analysis, we found that only folic acid supplementation after first trimester group had statistically remarkable relevance for respiratory allergic diseases risk. Wooldridge et al. (30) found that supplementation with methyl donors in the third trimester of pregnancy may increase the risk of allergy. All of the results suggested that methylation in early pregnancy may be useful but in the third trimester there may not be benefit and may be harmful.

Previous studies have reported the effect of folic acid on allergic diseases. One meta-analysis with only 5 included studies (9) showed no significant association between folic acid supplementation and asthma during early pregnancy (RR = 1.01, 95% CI = 0.78–1.30). However, this study may lead to low reliability of results due to limited included studies. Moreover, no other stratified analysis results were observed in this meta-analysis because of the small number of literatures. The other recent meta-analysis with 10 studies (31) showed that maternal folate intake during pregnancy is significantly related to the risk of infant asthma. In addition, the dose-response relationship in this study showed that the risk effect of folic acid supplements on allergic diseases increased with the increasing of folic acid exposure dose. However, this result was unreliable because they used only two articles to perform dose-response analysis. By contrast, 15 references with different exposure periods (First trimester vs. After first trimester vs. Whole pregnancy) were included in the present meta-analysis, thus providing it a large sample size. Hence, our conclusion is credible.

In this study, the significant association between the use of folic acid supplements and the increased risk of allergic diseases in infants and young children was found only when the dose was <400 μg/day. Moreover, meta-regression analyses showed that the risk effect of folic acid supplements on allergic diseases decreased with the increasing of folic acid exposure dose. All of these results suggested that pregnant women should increase the intake of folic acid supplements during pregnancy. Roy et al. (25) reported that high plasma folate in mid-pregnancy was associated with decreased odds of wheezing at age 3 (OR = 0.67, 95% CI = 0.46–0.97). In addition, Molloy et al. (27) reported that high doses of folic acid supplementation were not associated with eczema in the offspring (RR = 0.97, 95% CI = 0.67–1.38). However, take into account that most low dose studies were performed in the countries without folic acid fortification, pregnant women only used folic acid supplements not folic acid fortification foods may be the major reason.

In some western countries, giving folic acid to expectant mothers has been found to reduce the risk of neural tube malformation (NTD). In 1991, the British medical research council published a study showing that taking folic acid before pregnancy reduced the risk of NTD in infants by 72%. In 1998, the United States mandated a folic acid fortification program for cereal products, and Australia officially started its folate fortification program in 2009. There are now 80 countries with similar policies, and the prevalence of NTD decreased significantly. Meanwhile, many European countries did not have mandatory folic acid fortification policies, they only recommend folic acid supplementation during pregnancy to reduce the incidence of NTD in infants. Khoshnood et al. (32) showed that there has been no substantial decline in NTD prevalence in Europe over the past 20 years, despite a long-standing recommendation in European countries that women take folic acid supplements during pregnancy. Interestingly, stratified analyses in our study revealed that the association between the use of folic acid supplements during pregnancy and the increased risk of allergic diseases in infants and young children was significant only in the countries without folic acid fortification such as Europe. In addition, the risk of allergic diseases dramatically increased when pregnant women only used folic acid supplements. In light of that there has been no substantial decline in NTD prevalence in Europe over the past 20 years because of low intake of folic acid, folate fortification program should be performed to increase the intake dose of folic acid.

Nonetheless, the meta-analysis has some limitations. This study focused on the relationship between folic acid intake and allergic diseases. By contrast, a number of literatures has linked other nutrients with allergic diseases, including vitamin D, probiotics, and prebiotics and so on. The results of this meta-analysis were further limited by different exposure dose, different exposure period, and different exposure type; these parameters can alter the relationship between folic acid and risks of allergic diseases. In the present meta-analysis, we extracted the RR values adjusted by possible confounding factors such as feeding, family history and so on and performed subgroup analysis to exclude the bias factors. Moreover, only few studies reported the differences among different serum folate level. Therefore, it is not possible to perform sub-group analysis to clarify the difference between synthetic folic acid and serum folate in the review. In addition, randomized clinical trials were not included due to ethical issues.

Folic acid plays an important role in the formation of neonatal cardio-cerebrovascular system and is recommended for consumption as oral supplements or other forms for pregnant mothers in various countries. Our results suggested that folic acid intake can be a risk factor for allergic diseases, especially respiratory tract allergies among infants and young children. Furthermore, pregnant women should pay attention to supplementation of folic acid from both folic acid supplements and fortified foods with folic acid during pregnancy.

## Data Availability Statement

The raw data supporting the conclusions of this article will be made available by the authors, without undue reservation.

## Author Contributions

ZC and DM designed this manuscript. ZC and YX selected articles for inclusion, extracted data, and assessed risk of bias. XY and YD planned the statistical analyses. ZC wrote the first draft of the paper and all authors revised it critically for important intellectual content. All authors have read and approved the final manuscript.

## Conflict of Interest

The authors declare that the research was conducted in the absence of any commercial or financial relationships that could be construed as a potential conflict of interest.

## References

[1] Barry Kay A. Allergy and hypersensitivity: history and concepts. In: Barry Kay A Kaplan AP Bousquet J Holt PG, editors. Allergy and Allergic Diseases. New York, NY: Wiley (2008). p. 1–22.

[2] Miyake Y Sasaki S Tanaka K Hirota Y. Maternal B vitamin intake during pregnancy and wheeze and eczema in Japanese infants aged 16–24 months: the Osaka maternal and child health study. J. Pediatric Allergy Immunol. (2011) 22:69–74. 10.1111/j.1399-3038.2010.01081.x 20561231

[3] Crane J von Mutius E Custovic A Epidemiology of allergic disease. In: Holgate ST Church MK Lichtenstein LM editors. Allergy. 3rd ed. Edinburgh: Mosby (2006). p. 233–46. 10.1016/B978-0-323-03227-8.50019-6

[4] Daly LE Kirke PN Molloy A Weir DG Scott JM. Folate levels and neural tube defects. Implications for prevention. JAMA. (1995) 274:1698–702. 10.1001/jama.274.21.1698 7474275

[5] Bergström L Nutrient losses and gains in the preparation of foods: NLG project. Rapport-Livsmedelsverket. (1996) 57:77–8. 10.1016/0308-8146(96)89017-0

[6] Czeizel AE Dudás I Paput L Bánhidy F. Prevention of neural-tube defects with periconceptional folic acid, methylfolate, or multivitamins? Ann. Nutr. Metab. (2011) 58:263–71. 10.1159/000330776 21865678

[7] Obeid R Holzgreve W Pietrzik K. Is 5-methyltetrahydrofolate an alternative to folic acid for the prevention of neural tube defects? J. Perinat. Med. (2013) 41:469–83. 10.1515/jpm-2012-0256 23482308

[8] Scaglione F Panzavolta G. Folate, folic acid and 5-methyltetrahydrofolate are not the same thing. Xenobiotica. (2014) 44:480–8. 10.3109/00498254.2013.845705 24494987

[9] Crider KS Cordero AM Qi YP Mulinare J Dowling NF Berry RJ. Prenatal folic acid and risk of asthma in children: a systematic review and meta-analysis. Am. J. Clin. Nutr. (2013) 98:1272–81. 10.3945/ajcn.113.065623 24004895 PMC5369603

[10] Dennis RJ. Has mandatory folic acid supplementation of foods increased the risk of asthma and allergic disease? J. Allergy Clin. Immunol. (2009) 123:1260–61. 10.1016/j.jaci.2009.04.023 19447481

[11] Haberg SE London SJ Stigum H Nafstad P Nystad W. Folic acid supplements in pregnancy and early childhood respiratory health. Arch. Dis. Child. (2009) 94:180–4. 10.1136/adc.2008.142448 19052032 PMC3612898

[12] Dunstan JA West C McCarthy S Metcalfe J Meldrum S Oddy WH. The relationship between maternal folate status in pregnancy, cord blood folate levels, and allergic outcomes in early childhood. Allergy. (2012) 67:50–7. 10.1111/j.1398-9995.2011.02714.x 21923665

[13] Bekkers MB Elstgeest LE Scholtens S Haveman-Nies A de Jongste JC Kerkhof M. Maternal use of folic acid supplements during pregnancy, and childhood respiratory health and atopy. Eur. Respir. J. (2012) 39:1468–74. 10.1183/09031936.00094511 22034647

[14] Moher D Liberati A Tetzlaff J Altman DG PRISMA Group. Reprint—preferred reporting items for systematic reviews and meta-analyses: the PRISMA statement. Phys Ther. (2009) 89:873–80. 19723669

[15] Wells G The Newcastle-Ottawa Scale (NOS) for assessing the quality of nonrandomised studies in meta-analyses. In: Symposium on Systematic Reviews: Beyond the Basics. Oxford (2000).

[16] Granell R Heron J Lewis S Davey Smith G Sterne JA Henderson J. The association between mother and child MTHFR C677T polymorphisms, dietary folate intake and childhood atopy in a population-based, longitudinal birth cohort. Clin. Exp. Allergy. (2008) 38:320–8. 10.1111/j.1365-2222.2007.02902.x 18070159

[17] Whitrow MJ Moore VM Rumbold AR Davies MJ. Effect of supplemental folic acid in pregnancy on childhood asthma: a prospective birth cohort study. Am. J. Epidemiol. (2009) 170:1486–93. 10.1093/aje/kwp315 19880541

[18] Magdelijns FJ Mommers M Penders J Smits L Thijs C. Folic acid use in pregnancy and the development of atopy, asthma, and lung function in childhood. Pediatrics. (2011) 128:e135–144. 10.1542/peds.2010-1690 21690114

[19] Kiefte-de Jong JC Timmermans S Jaddoe VW Hofman A Tiemeier H Steegers EA. High circulating folate and vitamin B-12 concentrations in women during pregnancy are associated with increased prevalence of atopic dermatitis in their offspring. J. Nutr. (2012) 142:731–8. 10.3945/jn.111.154948 22399526

[20] Martinussen MP Risnes KR Jacobsen GW Bracken MB. Folic acid supplementation in early pregnancy and asthma in children aged 6 years. Am. J. Obstet. Gynecol. (2012) 206:72.e71–77. 10.1016/j.ajog.2011.07.033 21982024 PMC3246127

[21] Zetstra-van der Woude PA De Walle HE Hoek A Bos HJ Boezen HM Koppelman GH. Maternal high-dose folic acid during pregnancy and asthma medication in the offspring. Pharmacoepidemiol. Drug. Saf. (2014) 23:1059–65. 10.1002/pds.3652 24930442

[22] Veeranki SP Gebretsadik T Mitchel EF Tylavsky FA Hartert TV Cooper WO. Maternal folic acid supplementation during pregnancy and early childhood asthma. Epidemiology. (2015) 26:934–41. 10.1097/EDE.0000000000000380 26360371 PMC4900760

[23] Parr CL Magnus MC Karlstad O Haugen M Refsum H Ueland PM. Maternal folate intake during pregnancy and childhood asthma in a population-based cohort. Am. J. Respir. Crit. Care Med. (2017) 195:221–8. 10.1164/rccm.201604-0788OC 27518161 PMC5394786

[24] den Dekker HT Jaddoe VWV Reiss IK de Jongste JC Duijts L. Maternal folic acid use during pregnancy, methylenetetrahydrofolate reductase gene polymorphism, and child's lung function and asthma. Clin. Exp. Allergy. (2018) 48:175–85. 10.1111/cea.13056 29117460

[25] Roy A Kocak M Hartman TJ Vereen S Adgent M Piyathilake C. Association of prenatal folate status with early childhood wheeze and atopic dermatitis. Pediatr. Allergy Immunol. (2018) 29:144–50. 10.1111/pai.12834 29168294 PMC6087709

[26] Trivedi MK Sharma S Rifas-Shiman SL Camargo CA Jr Weiss ST Oken E. Folic acid in pregnancy and childhood asthma: a US cohort. Clin. Pediatr. (2018) 57:421–7. 10.1177/0009922817729482 28884603 PMC5823746

[27] Molloy J Collier F Saffery R Allen KJ Koplin JJ Louise Ponsonby A. Folate levels in pregnancy and offspring food allergy and eczema. Pediatr. Allergy Immunol. (2020) 31:38–46. 10.1111/pai.13128 31566807

[28] Dolinoy DC. Nutrition and epigenetics: an interplay of dietary methyl donors, one-carbon metabolism and DNA methylation. J. Nutr. Biochem. (2012) 23:853–9. 10.1016/j.jnutbio.2012.03.003 22749138 PMC3405985

[29] Hollingsworth JW Maruoka S Boon K Garantziotis S Li Z Tomfohr J. *In utero* supplementation with methyl donors enhances allergic airway disease in mice. J. Clin. Invest. (2016) 126:2012. 10.1172/JCI87742 27135881 PMC4855943

[30] Wooldridge AL Bischof RJ Liu H Heinemann GK Gatford KL. Late gestation maternal dietary methyl donor and cofactor supplementation in sheep partially reverses protection against allergic sensitization by IUGR. Am. J. Physiol. (2017) 314:22–3. 10.1152/ajpregu.00549.2016 28978515 PMC5866368

[31] Li W Xu B Cao Y Shao Y Wu W Zhou J. Association of maternal folate intake during pregnancy with infant asthma risk. Sci. Rep. (2019) 9:8347. 10.1038/s41598-019-44794-z 31171831 PMC6554315

[32] Khoshnood B Loane M de Walle H Arriola L Addor MC Barisic I. Long term trends in prevalence of neural tube defects in Europe: population based study. Bmj. (2015) 351:h5949. 10.1136/bmj.h5949 26601850 PMC4658393

